# Supplementary material for: Darkness promotes dishonesty in a coin toss task: A pre-registered conceptual replication of Experiment 1 of Zhong, Bohns, and Gino (2010)
Source: PLoS One. 2023 Dec 21;18(12):e0294484. doi: 10.1371/journal.pone.0294484 (PMC10734923; doi:10.1371/journal.pone.0294484)
Supplement: Attachment — Submitted filename: Response to Reviewers.docx [file pone.0294484.s001.docx]

Dr. Guy Hochman

Academic Editor

*PLOS ONE*

23, October 2023

Dear Dr. Hochman,

Thank you very much for handling our manuscript titled, “**Darkness promotes dishonesty in a coin toss task: A pre-registered conceptual replication of Experiment 1 of Zhong, Bohns, and Gino (2010)**” (PONE-D-23-27810). We have revised the manuscript according to your and the reviewers’ helpful comments to improve the quality of the manuscript.

Dear Reviewers:

Thank you for the constructive comments and positive evaluations. It is truly encouraging and serves as a great motivation for us to continue our work. We have improved our manuscript based on your comments. Below, we have outlined our point-by-point responses.

**# Journal Requirements**

*When submitting your revision, we need you to address these additional requirements.*

1) *The Please ensure that your manuscript meets PLOS ONE's style requirements, including those for file naming. The PLOS ONE style templates can be found at　https://journals.plos.org/plosone/s/file?id=wjVg/PLOSOne_formatting_sample_main_body.pdf and　https://journals.plos.org/plosone/s/file?id=ba62/PLOSOne_formatting_sample_title_authors_affiliations.pdf.*

**Reply:** We have carefully checked the template you provided and made necessary adjustments to the manuscript in accordance with it. We have also labeled the files in this submission as you asked.

2) *Please expand the acronym “JST SPRING” (as indicated in your financial disclosure) so that it states the name of your funders in full. This information should be included in your cover letter; we will change the online submission form on your behalf.*

**Reply:** The full designation for 'JST SPRING' stands for the 'Japan Science and Technology Agency, Support for Pioneering Research Initiated by the Next Generation'. Furthermore, the revised Financial Disclosure Statement is as follows:

**“This work was supported by Japan Science and Technology Agency, Support for Pioneering Research Initiated by the Next Generation (https://www.jst.go.jp/jisedai/en/index.html), Grant Number JPMJSP2136 (HL), Grants-in-Aid for Scientific Research of the Japan Society for the Promotion of Science (https://www.jsps.go.jp/j-grantsinaid/index.html), Grant Numbers 22K18263, 21H03784, and 20H04581(YY). The funders had no role in the study design, data collection and analysis, publication decision, or manuscript preparation.”**

3) *Thank you for stating the following in the Acknowledgments Section of your manuscript: "We would like to thank Yanqing Tang, Linzi Liu for the assistance in preparing ethical review documents and data collection. This study was financially supported by JST SPRING (https://www.jst.go.jp/jisedai/en/index.html), Grant Number JPMJSP2136 (HL), Grants-in-Aid for Scientific Research of the Japan Society for the Promotion of Science (https://www.jsps.go.jp/j-grantsinaid/index.html), Grant Numbers 22K18263, 21H03784, and 20H04581 (YY). The funders had no role in the study design, data collection and analysis, publication decision, or manuscript preparation."*

*We note that you have provided funding information that is not currently declared in your Funding Statement. However, funding information should not appear in the Acknowledgments section or other areas of your manuscript. We will only publish funding information present in the Funding Statement section of the online submission form.*

*Please remove any funding-related text from the manuscript and let us know how you would like to update your Funding Statement. Currently, your Funding Statement reads as follows: "This study was financially supported by JST SPRING (https://www.jst.go.jp/jisedai/en/index.html), Grant Number JPMJSP2136 (HL), Grants-in-Aid for Scientific Research of the Japan Society for the Promotion of Science (https://www.jsps.go.jp/j-grantsinaid/index.html), Grant Numbers 22K18263, 21H03784, and 20H04581 (YY). The funders had no role in the study design, data collection and analysis, publication decision, or manuscript preparation."*

*Please include your amended statements within your cover letter; we will change the online submission form on your behalf.*

**Reply:** We deeply regret the oversight on my part. We have amended the acknowledgements section of the manuscript as directed, deleted all text related to funding. The amended statements were attached to Reply 2) above. Thank you.

4) *We note that you have stated that you will provide repository information for your data at acceptance. Should your manuscript be accepted for publication, we will hold it until you provide the relevant accession numbers or DOIs necessary to access your data. If you wish to make changes to your Data Availability statement, please describe these changes in your cover letter and we will update your Data Availability statement to reflect the information you provide.*

**Reply:** Thank you for bringing this to my attention. We have revised the content of the statement and incorporated a link that can access the data. The updated statement reads as follows: **“The datasets analyzed in this study are available from the OSF repository (osf.io/bvqsn).”**

5) *Your ethics statement should only appear in the Methods section of your manuscript. If your ethics statement is written in any section besides the Methods, please move it to the Methods section and delete it from any other section. Please ensure that your ethics statement is included in your manuscript, as the ethics statement entered into the online submission form will not be published alongside your manuscript.*

**Reply:** Thank you for drawing my attention to this matter. I have incorporated the ethics statement into the methods section and duly removed it from other section.

6) *We notice that your supplementary figures are uploaded with the file type 'Figure'. Please amend the file type to 'Supporting Information'. Please ensure that each Supporting Information file has a legend listed in the manuscript after the references list.*

**Reply:** My sincerest apologies for any confusion. It appears there may have been a misunderstanding on my part regarding your publication's guidelines. I would like to clarify that these are “figures” not “supplementary figures”. Consequently, I have made appropriate revisions to the manuscript to reflect this. They will be uploaded with the file type “Figure” in this submission. Should you consider it would be more fitting for them to be submitted as supplementary figures or have any other concerns, please do not hesitate to inform me. I am more than willing to make further adjustments as necessary.

7) *Please upload a copy of Figure 1 and 2, to which you refer in your text on page 6 and 9. If the figure is no longer to be included as part of the submission please remove all reference to it within the text.*

**Reply:** As previously mentioned, I mistakenly submitted Figure 1 and 2 as supplementary figures. I deeply regret any confusion caused. I have addressed this in the revised manuscript.

8) *Please include a separate caption for each figure in your manuscript.*

**Reply:** I apologize again for the oversight that submitted the figures as supplementary figures. I have ensured the revised manuscript meets your journal's requirements concerning figure presentation. Specifically, the misplaced captions have been removed from the end of the manuscript and have been appropriately positioned where Figures 1 and 2 are first cited.

9) *Please review your reference list to ensure that it is complete and correct. If you have cited papers that have been retracted, please include the rationale for doing so in the manuscript text, or remove these references and replace them with relevant current references. Any changes to the reference list should be mentioned in the rebuttal letter that accompanies your revised manuscript. If you need to cite a retracted article, indicate the article’s retracted status in the References list and also include a citation and full reference for the retraction notice.*

**Reply:** We have diligently reviewed and adjusted the reference list to meet the standards of your journal. To the best of our knowledge, there are no retracted papers.

**# Review Comments to the Author**

**Reviewer #1:** *This seems to me a good replication study with pre-registration, power analysis, and quantifiable manipulation of light/darkness condition. The use of the dice task also extends generalizability. The absence of the anonymity difference does not seem too surprising to me -- I would expect perceived anonymity to be likely sensitive to experimental setup and the presence and positioning of other individuals. It would be helpful for the authors to report means and SDs in addition to statistical tests so we can see whether there might be floor or ceiling effects. Overall I think this is a worthy effort that should be published.*

**Reply:** Thank you for your positive feedback. We are gratified to note that you recognize the importance and implications of our study. Additionally, we are in agreement with your insights on anonymity. Per your recommendation, we have provided further details to the readers in the results section, such as means and *SDs*.

***“The mean score reported in dark condition was 5.96 (SD = 1.48; Mdn = 6); the mean score reported in light condition was 5.39 (SD = 1.56; Mdn = 6).”***

***“On the other hands, the mean perceived anonymity in dark condition was 4.37 (SD = 0.87), while that in light condition was 4.24 (SD = 1.11).”***

**Reviewer #2:** *In the first line of the introduction, it is asserting that dishonesty is pervasive. Is it? Most bad and socially disapproved behavior is not normally distributed (e.g., Kim B. Serota et al.’s numerous studies of the few prolific liars). I also noted that in the current data, there did not appear to be pervasive cheating.*

**Reply:** As you rightly pointed out, our phrasing could be misunderstandings. We initially aimed to convey that dishonest behavior often occurs in daily life. We have revised the opening sentence of the introduction to ensure clarity and avoid potential misunderstandings, ***“The potential adverse consequences of dishonesty in daily life have catalyzed extensive research in psychology”***. We deeply appreciate your insightful comments, and we're grateful for the information you provided.

**Reviewer #2:** *It would be helpful to report both means and standard deviations in text especially for the number of tails.*

**Reply:** Thank you for your feedback, which guided us to enhance our presentation of results. In response, we have provided the means and standard deviations of the number of tails in the revised manuscript.

***“The mean score reported in dark condition was 5.96 (SD = 1.48; Mdn = 6); the mean score reported in light condition was 5.39 (SD = 1.56; Mdn = 6).”***

**Reviewer #2:** *Why are the means different but not the medians in the tails. Do outliers explain the results?*

**Reply:** That is an interesting question. We consider that the reason lies in the outliers as you have mentioned, particularly those participants reporting high tails. The distributions of the individual data show that there were more participants in the dark condition who reported high tails. Precisely, that was the central conclusion of the experiment. Conversely, data from the light condition predominantly clustered around the value of 6, which makes a lower Mean. We added this explanation in the text.

***“It is evident that the significant difference was found in means, but not in the medians. This discrepancy can be attributed to that some participants reported fairly higher scores (> 7) in the dark condition, whereas this was not the case in the light condition, with participants reporting scores that widely dispersed around 6. This indicates that dishonesty was more prevalent in the dark condition.”***

**Reviewer #2:** *The difference in findings between means and medians and the overall level/extent of cheating should be mentioned in the discussion section.*

**Reply:** We greatly appreciate your insightful comment. In response, we have revised the manuscript to incorporate the content aligned with your suggestions.

***“It is evident that the significant difference was found in means, but not in the medians. This discrepancy can be attributed to that some participants reported fairly higher scores (> 7) in the dark condition, whereas this was not the case in the light condition, with participants reporting scores that widely dispersed around 6. This indicates that dishonesty was more prevalent in the dark condition.”***

***“Moreover, taken as a whole, dishonest behavior is evident at a moderate level.”***

**Reviewer #2:** *I really liked the simplicity of the experiment.*

**Reply:** We deeply appreciate your positive comment; it's truly our privilege.

The manuscript has been revised according to these replies. We are very grateful for all reviewers’ efforts and comments on this manuscript. If there is still any problem, please feel free and let us know.

Sincerely,

Huanxu Liu

Graduate School of Human-Environment Studies, Kyushu University,

744 Motooka, Nishi-ku, Fukuoka 819-0395 Japan

E-mail: ryuukansyo@gmail.com

TEL & FAX: +81-92-802-5837

Yuki Yamada, Ph.D.

Faculty of Arts and Science, Kyushu University,

744 Motooka, Nishi-ku, Fukuoka 819-0395, Japan

E-mail: yamadayuk@gmail.com

TEL & FAX: +81-92-802-5837
