# Supplementary material for: Darkness promotes dishonesty in a coin toss task: A pre-registered conceptual replication of Experiment 1 of Zhong, Bohns, and Gino (2010)
Source: PLoS One. 2023 Dec 21;18(12):e0294484. doi: 10.1371/journal.pone.0294484 (PMC10734923; doi:10.1371/journal.pone.0294484)
Supplement: Attachment — Submitted filename: Response to Reviewers.docx [file pone.0294484.s002.docx]

Dr. Guy Hochman

Academic Editor

*PLOS ONE*

31, October 2023

Dear Dr. Hochman,

Thank you very much for handling our manuscript titled, “**Darkness promotes dishonesty in a coin toss task: A pre-registered conceptual replication of Experiment 1 of Zhong, Bohns, and Gino (2010)**” (PONE-D-23-27810R1). We have revised the manuscript according to your comments to improve the quality and to increase the impact of our paper.

We are very grateful for your efforts and comments on this manuscript. If there is still any problem, please feel free and let us know.

Below, we have outlined our point-by-point responses.

**# Editor Comments**

1) *First, on page 3, you refer to physiology research in dishonesty but only cite one source (that deals with hormones). Please add 1-2 papers that used physiological measures to study cheating and immoral behavior (whichever you like).*

**Reply:** We have incorporated the following references (about hormones and facial thermal variations) and subsequently refined the manuscript accordingly: ***“Physiological psychology has also contributed to the understanding of this issue, with the studies about the relation between physiological indicators (such as hormones and facial thermal variations) and dishonesty behavior [6, 7, 8]”***

***“7. Wibral M, Dohmen T, Klingmüller D, Weber B, Falk A. Testosterone Administration Reduces Lying in Men. PLoS ONE. 2012;7(10): e46774. https://doi.org/10.1371/journal.pone.0046774”***

***“8. Rajoub BA, Zwiggelaar R. Thermal facial analysis for deception detection. IEEE Trans Inf Forensics Secur. 2014;9(6): 1015-1023.　https://doi.org/10.1109/TIFS.2014.2317309.”***

2) *Second, when citing Zhang et al on page 4, l. 74-75, please add the page number to the end of the citation.*

**Reply:** Thank you for noting the problem. The specific page numbers have now been added.

**“*They said that “although the room in the experimental condition was darker than the one in the control condition, participants had no trouble seeing and identifying each other” [9, p. 314].”***

3) *Third, referring to the second reason you state for replicating Zhang et al., please explain why it is important to examine perceived anonymity (how it adds to what is already known in the literature). Moreover, please try to emphasize your unique contribution to the literature on cheating behavior (as it is not clear and explicit enough).*

**Reply:** Concerning the rationale for examining perceived anonymity, we have integrated the following sentences into the original manuscript. **“*Zhong et al. (2010) did not examine any measures related to perceived anonymity, thus it is unclear whether the mechanism in which room darkness leads to cheating really involves perceived anonymity. That is, the effect of anonymity has insufficient plausible grounds to support the conclusions of the original study. Further empirical evidence is required to substantiate this claim.”***

Concurrently, we have further underscored our strengths.

**“*In summation, our refinements provide several key advantages: first, they can control the variability of actual anonymity. Second, they can provide evidence for the first time on perceived anonymity in the observed phenomenon. Third, they facilitate a discussion regarding the potential generalizability of the phenomenon, especially under tasks more suitable for detecting dishonest behavior.”***

4) *Finally, please refer to up-to-date research when interpreting your results and develop the argument about the CMT. You might benefit from using the following references (but don’t feel obligated to use them). But please try your best to better explain why you find the difference between the light and dark conditions and how it relates to recent literature in behavioral ethics.*

**Reply:** Thank you very much for the information! Addressing the issues you point out, we have enriched our discussion section by citing the recent research you provided. ***“A further alternative view of the relation between darkness and dishonesty can be offered by the latest findings from social psychology. It has been reported that the effect of brightness on pro-social actions may be moderated by societal factors, such as self-construal [25]. Moreover, the influence of brightness on pro-social behavior is believed to be mediated, in part, by satisfaction with illumination and perceived anonymity [26]. Intriguingly, the findings on perceived anonymity by Ru et al. (2022) differ from those of the present study. They observed that variations in brightness affected participants’ perceived anonymity. It is postulated that this discrepancy may arise from the subtle differences in the design of the laboratory environment and the positioning of the experimenter. However, it is important to note that although pro-social actions and dishonest behavior are often considered in tandem within behavioral ethics, their subtle distinctions (e.g., dishonest behavior frequently entails the possibility of more stringent penalties)*** ***make it debatable to what extent findings on pro-social behavior can be reliably applied to dishonest actions. In summary, these suggest the presence of intricate underlying mechanisms and multifaceted phenomena awaiting exploration in subsequent studies.”***

**# Journal Requirements**

1) *Please review your reference list to ensure that it is complete and correct. If you have cited papers that have been retracted, please include the rationale for doing so in the manuscript text, or remove these references and replace them with relevant current references. Any changes to the reference list should be mentioned in the rebuttal letter that accompanies your revised manuscript. If you need to cite a retracted article, indicate the article’s retracted status in the References list and also include a citation and full reference for the retraction notice.*

**Reply:** We have diligently reviewed and adjusted the reference list to meet the standards of *PLOS ONE*. To the best of our knowledge, there are no retracted papers.

The manuscript has been revised according to these replies. We are very grateful for your efforts and comments on this manuscript. If there is still any problem, please feel free and let us know.

Sincerely,

Huanxu Liu

Graduate School of Human-Environment Studies, Kyushu University,

744 Motooka, Nishi-ku, Fukuoka 819-0395 Japan

E-mail: ryuukansyo@gmail.com

TEL & FAX: +81-92-802-5837

Yuki Yamada, Ph.D.

Faculty of Arts and Science, Kyushu University,

744 Motooka, Nishi-ku, Fukuoka 819-0395, Japan

E-mail: yamadayuk@gmail.com

TEL & FAX: +81-92-802-5837
